# CYB5D2 Requires Heme-Binding to Regulate HeLa Cell Growth and Confer Survival from Chemotherapeutic Agents

**DOI:** 10.1371/journal.pone.0086435

**Published:** 2014-01-22

**Authors:** Anthony Bruce, Adrian P. Rybak

**Affiliations:** 1 Medical Sciences Program, Faculty of Health Sciences, McMaster University, Hamilton, Ontario, Canada; 2 Father Sean O’Sullivan Research Institute, Hamilton, Ontario, Canada; 3 Hamilton Centre for Kidney Research (HCKR), St. Joseph’s Hospital, Hamilton, Ontario, Canada; Columbia University, United States of America

## Abstract

The cytochrome b5 domain containing 2 (CYB5D2; Neuferricin) protein has been reported to bind heme, however, the critical residues responsible for heme-binding are undefined. Furthermore, the relationship between heme-binding and CYB5D2-mediated intracellular functions remains unknown. Previous studies examining heme-binding in two cytochrome b5 heme-binding domain-containing proteins, damage-associated protein 1 (Dap1; *Saccharomyces cerevisiae*) and human progesterone receptor membrane component 1 (PGRMC1), have revealed that conserved tyrosine (Y) 73, Y79, aspartic acid (D) 86, and Y127 residues present in human CYB5D2 may be involved in heme-binding. CYB5D2 binds to type *b* heme, however, only the substitution of glycine (G) at D86 (D86G) within its cytochrome b5 heme-binding (cyt-b5) domain abolished its heme-binding ability. Both CYB5D2 and CYB5D2(D86G) localize to the endoplasmic reticulum. Ectopic CYB5D2 expression inhibited cell proliferation and anchorage-independent colony growth of HeLa cells. Conversely, CYB5D2 knockdown and ectopic CYB5D2(D86G) expression increased cell proliferation and colony growth. As PGRMC1 has been reported to regulate the expression and activities of cytochrome P450 proteins (CYPs), we examined the role of CYB5D2 in regulating the activities of CYPs involved in sterol synthesis (CYP51A1) and drug metabolism (CYP3A4). CYB5D2 co-localizes with cytochrome P450 reductase (CYPOR), while CYB5D2 knockdown reduced lanosterol demethylase (CYP51A1) levels and rendered HeLa cells sensitive to mevalonate. Additionally, knockdown of CYB5D2 reduced CYP3A4 activity. Lastly, CYB5D2 expression conferred HeLa cell survival from chemotherapeutic agents (paclitaxel, cisplatin and doxorubicin), with its ability to promote survival being dependent on its heme-binding ability. Taken together, this study provides evidence that heme-binding is critical for CYB5D2 in regulating HeLa cell growth and survival, with endogenous CYB5D2 being required to modulate CYP activities.

## Introduction

Progesterone receptor membrane component 1 (PGRMC1) is the most extensively investigated member of the membrane associated progesterone receptor (MAPR) family. The PGRMC1 protein is reported to have multiple functions including steroid signaling, sterol synthesis, cytochrome P450 activation and drug metabolism [1]–[3]. The yeast homolog of PGRMC1, damage associated protein 1 (Dap1), a cytochrome b5 heme-binding (cyt-b5) protein, is required for survival from the DNA methylating agent, methyl methane-sulfonate (MMS) [4], [5]. Substitution of the conserved D91 residue with G prevents Dap1 from association with heme and Dap1(D91G) is incapable of protecting yeast from MMS-induced toxicity [6].

In accordance with PGRMC1 containing a cyt-b5 domain, PGRMC1 binds to heme and its association with heme contributes to its function [7]. UV-visible absorption and electron paramagnetic resonance (ESR) spectra were used to demonstrate that PGRMC1 binds to *b*-type heme [8]. The hydrophobic heme-binding pocket of PGRMCI was affected by mutating two conserved tyrosine (Y107, Y113) residues [8], while mutating the conserved aspartic acid residue at position 120 to glycine (D120G) in PGRMC1 resulted in the loss of its heme-binding ability [7].

Cytochrome b5 domain containing 2 (CYB5D2; Neuferricin) possesses similar structural components as PGRMC1, including an amino-terminal transmembrane (TM) domain and a conserved cyt-b5 domain [9]. Consistent with these structural similarities, human CYB5D2 was reported to bind heme and deletion of the cyt-b5 domain (amino acid residues 55–98) ablated this interaction [9]. Both proteins are 40.4% identical and 55.8% similar within their cyt-b5 domain [10]. It has been previously shown that deletion of the TM domain in CYB5D2 abolished its perinuclear localization, compromising its survival from etoposide treatment. Furthermore, deletion of the cyt-b5 domain (amino acid residues 35–134) of CYB5D2 increased the sensitivity of HeLa cells to etoposide toxicity [10].

Cytochrome b5 heme-binding (cyt-b5) domain-containing proteins have been shown to bind and regulate cytochrome P450 (CYP) proteins, heme-dependent monooxygenases which participate in xenobiotic detoxification, sterol and steroid biosynthesis, and metabolism of pharmaceutical drugs [11]. Dap1, a heme-binding protein in *Saccharomyces cerevisiae*, was initially shown to target and stabilize the CYP protein, Cyp51A1 (Erg11) [5], [6], and further shown to bind and positively regulate Cyp51A1 and Cyp61A1 (Erg5) [12]. The ability of Dap1 to regulate Cyp51A1 activity has been shown to occur in a heme-dependent fashion [6], [12]. Human PGRMC1 has been shown to bind CYP51A1 (lanosterol demethylase), with loss of PGRMC1 reducing CYP51A1 activity, impairing cholesterol synthesis and resulting in elevated lanosterol levels [12], [13]. In addition, PGRMC1 has been shown to bind other CYP enzymes (CYP3A4, CYP21A2, CYP7A1, CYP2C2 and CYP2C8) [12], [13], with the activities of CYP2C2 and drug metabolizing P450 cytochromes (CYP3A4 and CYP2C8) being modestly affected in human embryonic kidney (HEK293) and HepG2 liver cells [13]. Furthermore, the heme-binding defective mutant, PGRMC1(D120G), reduced the survival of MDA-MB-231 breast cancer cells from doxorubicin and camptothecin [7], implicating the heme-binding capacity of PGRMC1 in regulating susceptibility towards chemotherapeutic agents.

In the current study, we demonstrate that mutating the conserved aspartic acid residue at position 86 (D86) within the cyt-b5 domain of CYB5D2 ablates its heme-binding ability, but does not affect its endoplasmic reticulum (ER) localization. This heme-binding defective mutant (D86G) could not precipitate with hemin-agarose, unlike wild-type CYB5D2. Furthermore, the CYB5D2(D86G) mutant cannot be oxidized or reduced *in vitro*, unlike wild-type CYB5D2. While CYB5D2 expression inhibited HeLa cell proliferation and colony growth in soft agar, the CYB5D2(D86G) mutant increased the proliferation and anchorage-independent growth of HeLa cells. Similarly, knockdown of CYB5D2 increased HeLa cell proliferation and soft agar colony growth. Therefore, loss of heme-binding ability results in CYB5D2 “loss of function”, which seems to affect its putative tumor suppressor role. In addition, CYB5D2 co-localizes with cytochrome P450 reductase (CYPOR), a microsomal protein required for cytochrome P450 (CYP) protein activation. Moreover, loss of endogenous CYB5D2 reduced the activity of CYP proteins involved in sterol synthesis (CYP51A1) and drug metabolism (CYP3A4). Finally, knockdown of CYB5D2 and ectopic expression of heme-binding defective CYB5D2(D86G) increased the susceptibility of HeLa cells to paclitaxel, cisplatin and doxorubicin treatment. Taken together, heme-binding by CYB5D2 is required for cell growth and conferring survival from chemotherapeutic agents, with endogenous CYB5D2 being necessary to modulate CYP activities.

## Materials and Methods

### Cell Lines and Plasmids

HeLa and 293T cells were purchased from American Type Culture Collection (ATCC) and cultured in DMEM supplemented with 10% heat-inactivated fetal bovine serum (Sigma-Aldrich) and 1% penicillin/streptomycin (Life Technologies) according to ATCC instructions. Construction of vectors expressing amino-terminal HA-tagged CYB5D2, CYB5D2(ΔTM), and CYB5D2(Δcyt-b5) has been previously described [10]. CYB5D2(D86G), CYB5D2(Y73A), CYB5D2(Y79A), CYB5D2(Y127A) and CYB5D2 were inserted in amino-terminal HA- or FLAG-tagged pcDNA3-based vectors. The above mentioned mutants were generated by site-directed mutagenesis according to a published methodology [14].

Vectors expressing amino-terminal glutathione S-transferase (GST) fusion proteins, GST-CYB5D2 and GST-CYB5D2(D86G), were produced by insertion of CYB5D2(ΔTM) and CYB5D2(D86G-ΔTM) into pGEX2T. Primers and PCR conditions used to construct CYB5D2 mutants are summarized in Table S1.

### Purification of GST-CYB5D2 and GST-CYB5D2(D86G)

Fusion proteins of GST-CYB5D2 and GST-CYB5D2(D86G) were produced in the BL-21 *Escherichia coli* host using the pGEX2T/GST-CYB5D2 and pGEX2T/GST-CYB5D2(D86G) vectors following published conditions [14]. Thrombin (Sigma-Aldrich) was then used at a concentration of 1.5 µg/ml to cleave 1 mg of purified GST-CYB5D2 and GST-CYB5D2(D86G) protein present in the thrombin cleavage buffer [0.05 M Tris (pH 7.5), 0.15 M NaCl_2_, 2.5 mM CaCl_2_]. Thrombin cleavage was performed at room temperature for 6 hours (h) in order to cleave the recombinant GST from the CYB5D2 and CYB5D2(D86G) fusion proteins. GST-agarose was subsequently used for GST removal. The recombinant GST-free CYB5D2 and CYB5D2(D86G) proteins were confirmed by Western blot using our in-house generated anti-CYB5D2 rabbit polyclonal antibody [10].

### Analysis of Heme-binding Ability by CYB5D2

Association of CYB5D2 with heme/hemin was determined by several methods. An empty vector (pcDNA3.0) and pcDNA3-based vectors expressing amino-terminal FLAG-tagged CYB5D2, CYB5D2(Y73A), CYB5D2(Y79A), CYB5D2(D86G), CYB5D2(Y127A) were transiently expressed in 293T cells following calcium phosphate transfection, and allowed to express for 48 h. Cell lysates were prepared in a buffer containing 20 mM Tris (pH 7.4), 150 mM NaCl, 1 mM EDTA, 1 mM EGTA, 1% Triton X-100, 25 mM sodium pyrophosphate, 1 mM NaF, 1 mM β-glycerophosphate, 0.1 mM sodium orthovanadate, 1 mM PMSF, 2 μg/ml leupeptin and 10 μg/ml aprotinin. Hemin-agarose (Sigma-Aldrich) slurry was washed three times with co-immunoprecipitation buffer containing 0.1% Triton, 150 mM NaCl, 5 mM EDTA and 50 mM Tris (pH 7.5), followed by incubation of pre-washed hemin-agarose slurry (20 μl) with 100 μg of cell lysate at 4°C overnight with rotation. Hemin-agarose containing lysates were washed with 1 ml of co-immunoprecipitation buffer eight times prior to Western blot analysis with the indicated antibodies.

GST-CYB5D2 and GST-CYB5D2(D86G) (200 μg) were resuspended in 200 mM NaOH and 40% pyridine solution, to which 3 µl of 0.1 M K_3_Fe(CN)_6_ was added [15]. Protein solutions (at a final concentration of 200 μg/ml) were measured for absorbance by scanning from 350–490 nm wavelength at 1 nm increments using PRISM software on an Ultraspec2100 spectrophotometer (Thermo Fisher Scientific).

Heme-bound GST-CYB5D2 was also assayed by in-gel peroxidase reaction staining using a previously published protocol [16], [17]. Briefly, recombinant GST-CYB5D2 and GST-CYB5D2(D86G) (10 μg) were incubated with hemin (50 µM; Sigma-Aldrich) at room temperature for 30 minutes, followed by separation by SDS-PAGE without the addition of dithiothreitol (DTT). The SDS-PAGE gel was rinsed with 1×TBS for 5 min, followed by incubation with ECL Western Blotting Detection Kit (GE Healthcare) to develop a signal. Coomassie blue staining of the SDS-PAGE gel was carried out to verify equal protein loading.

### Determination of the Type of Heme that Binds to CYB5D2

Recombinant GST-free CYB5D2 and CYB5D2(D86G) proteins were dissolved in a buffer containing 200 mM NaOH, 40% pyridine and 3 µl of 0.1 M K_3_Fe(CN)_6_ (at a final concentration of 200 μg/ml) to determine their absorbance spectra under oxidizing conditions. To determine the absorbance spectra of CYB5D2 under reducing conditions, 2.5 mg/ml sodium dithionite (Sigma-Aldrich) [9], [18] was added to purified recombinant GST-free CYB5D2 and the absorbance was measured by scanning from 350–490 nm wavelength at 1 nm increments (Ultrospec 2100).

Association between heme and a heme-binding protein occurs through either a covalent (type *c* heme) or non-covalent (types *a* or *b* heme) interaction [19], with oxidized heme-bound (types *a, b* and *c*) proteins being distinguished by their absorbance peaks in the 500–600 nm range [15]. Under oxidizing conditions, recombinant GST-free CYB5D2 or CYB5D2(D86G) (200 μg/ml) were incubated with 20 µM hemin [20] and the absorbance spectra was recorded by scanning from 520–620 nm wavelength at 1 nm increments (Ultrospec 2100).

### Virus Production and Stable Cell Line Generation

In order to generate HeLa cells stably expressing CYB5D2(D86G), amino-terminal FLAG-tagged CYB5D2(D86G) was sub-cloned into the pLHCX vector. CYB5D2/pLHCX, CYB5D2(D86G)/pLHCX and empty vector (EV)/pLHCX constructs were each co-transfected into 293T cells along with VSV and GP retroviral packaging plasmids (Stratagene) using the calcium phosphate transfection method. Sixty hours post-transfection, the supernatants containing VSV-G pseudotyped, replication-incompetent retroviral particles were collected, filtered through a 0.45 µm filter and centrifuged for 2 h at 48,000 g. Viral pellets were resuspended in media containing 10 µg/ml polybrene (Sigma-Aldrich) and added to cells for 2 h, with periodic mixing at 20 min intervals, to allow for cell infection. Infection was selected in hygromycin (0.5 mg/ml; Life Technologies) to generate stable cell lines.

CYB5D2 shRNA (shCYB5D2) and non-specific shRNA control (shCTRL) lentiviral pool plasmids were purchased from Santa Cruz Biotechnology Inc., and packaged in 293T cells by co-transfecting each shRNA plasmid pool (10 µg) with plasmids (10 µg each) necessary for third-generation lentiviral production (pRSV-REV, pCMV-VSV-G, pMDLg/pRRE) [21], using the calcium phosphate transfection method. Filter-sterilized (0.45 µm) media containing shCYB5D2 and shCTRL lentivirus were subsequently concentrated by centrifugation for 2 h at 48,000 g, resuspended in 1 ml of DMEM media containing polybrene, and added to HeLa cells for 2 h, with periodic mixing at 20 min intervals, to allow for cell infection. Puromycin (1 µg/ml; Sigma-Aldrich) was subsequently added to select for infection.

### Anchorage-independent Growth Assay

HeLa stable cell lines (EV, CYB5D2, CYB5D2(D86G), shCTRL and shCYB5D2) were dissociated by trypsinization, and individualized (5×10^4^) cells were mixed with agarose-containing media and plated in 60 mm plates, as previously described [22]. Each experiment was conducted in triplicate. After 8 weeks, images were taken in 10 random fields per plate using the Zeiss Axiovert 200 M microscope (AxioVision 3.1 software) at 25X magnification, and the colonies consisting of ≥50 cells were counted. To determine colony size, images of spheres were captured at 50X magnification, and the mean diameter (in microns) of individual colonies was determined using ImagePro Plus 5.0 software (MediaCybernetics). Digital images of plates were taken using a Sony Cyber-shot (DSC-W220) digital camera (Sony Corporation) and subsequently processed using CorelDRAW Graphics Suite X4 software (Corel).

### Cell Proliferation Assay

To measure HeLa cell growth, 10^4^ cells were seeded in triplicate (12-well plates) in serum-supplemented DMEM media. Cells were trypsinized and counted with a haemocytometer using Trypan blue reagent (Sigma-Aldrich) to exclude for dead cells. Cells numbers were counted daily from day 1 to day 6. Cell proliferation assay was conducted in quadruplicate.

### Cell Viability Assays

Cell viability following chemotherapeutic drug and mevalonate treatment was measured using the Cell Proliferation Kit I (3-[4,5 dimethylthiazol-2-y]-2,5-diphenyl-tetrazolium bromide; MTT) assay (Roche). For MTT assays, cells were seeded at a density of 5×10^3^ cells per well in 96-well plates for 24 h. Cells were subsequently treated with either doxorubicin, cisplatin or paclitaxel (Sigma-Aldrich) at the indicated doses for 24 and 48 h. As a control, an equal volume of dimethylsulfoxide (DMSO) was administered for each stable cell line at each time point. Following treatment, cell viability was measured using the MTT assay, according to the manufacturer’s instructions. Absorbance readings were carried out on SPECTRAmax Plus 384 spectrophotometer (Molecular Devices).

For mevalonate treatments, cells were seeded at a density of 5×10^3^ cells per well in 96-well plates for 24 h, followed by aspirating media and re-feeding with DMEM +5% lipoprotein-deficient serum along with mevalonate (Sigma-Aldrich) at the indicated concentrations for 24 h. Following treatment, cell viability was measured using the MTT assay.

### Cytochrome P450 3A4 Activity Assay

Cytochrome P450 (CYP) 3A4 activity was measured using the CYP3A4 P450-Glo™ assay (with Luciferin-IPA) (Promega) as previously described [13]. Briefly, HeLa cell lines were seeded at a density of 2×10^4^ cells/well in 12-well plates (Sarstedt Inc.). Twenty-four hours later, 0.5 µg of CYPOR-YFP plasmid (kindly provided by Dr. Byron Kemper, University of Illinois, USA) was transfected into cells using Lipofectamine 2000 reagent (Life Technologies). Twenty-four hours after transfection, cells were washed with phosphate-buffered saline and fresh media containing luminogenic substrate (50 µM) was added. After a 3 h incubation period, 100 µl of media from each well was mixed with an equal volume of Luciferin Detection reagent and luminescence was measured using a LUMIstar luminometer (BMG LABTECH).

### Immunofluorescence

Immunofluorescent staining of cells was performed as previously described [22]. Briefly, HeLa cells were seeded in 8-well chamber slides (Thermo Fisher Scientific) for 24 h, fixed with 4% paraformaldehyde and permeabilized using 0.3% Triton X-100 in 1×PBS solution, and subsequently incubated with mouse anti-FLAG (M2; 1∶100, Sigma-Aldrich) and rabbit anti-GRP78 (H-129; Santa Cruz Biotechnology) antibodies. For co-staining of CYB5D2 and CYPOR, CYB5D2-expressing HeLa cells were seeded in a 60-mm tissue culture plate (Sarstedt) and transfected with CYPOR-YFP plasmid (10 µg) using Lipofectamine 2000 reagent (Life Technologies) according to the manufacturer’s instructions. Twenty four hours post-transfection, CYPOR-transfected cells were seeded in 8-well chamber slides and 24 h later, cells were fixed, permeabilized and incubated with mouse anti-FLAG antibody (M2). Slides were subsequently washed in 1×PBS solution and incubated with donkey anti-mouse IgG-FITC, donkey anti-rabbit IgG-FITC or donkey anti-rabbit IgG-Rhodamine secondary antibodies (1∶200 dilution; Jackson Laboratories) for 1 h, as required. Slides were mounted with a coverslip using Vectashield Mounting Medium with DAPI (Vector Laboratories, Inc., H-1200), and images were captured using a Zeiss Axiovert 200 M inverted fluorescence microscope using AxioVision 3.1 software. Images were processed using ImageJ software (version 1.43 u; W. Rasband, National Institute of Health).

### Western Blot Analysis

Whole cell homogenate lysates were prepared in a buffer containing 20 mM Tris (pH 7.4), 150 mM NaCl, 1 mM EDTA, 1 mM EGTA, 1% Triton X-100, 25 mM sodium pyrophosphate, 1 mM NaF, 1 mM β-glycerophosphate, 0.1 mM sodium orthovanadate, 1 mM PMSF, 2 μg/ml leupeptin and 10 μg/ml aprotinin. A total of 50 μg of whole cell lysate was separated by SDS-PAGE and transferred onto Immobilon-P membranes (Millipore). Membranes were blocked with 5% skim milk in 1× Tris-buffered saline containing 0.1% Tween-20 (TBST) solution and then incubated with the indicated antibodies at 4°C overnight. Primary antibodies and concentrations used were as follows: anti-CYP51A1 (13–431, 1∶1000; Proteintech Group, Inc.), anti-FLAG (M2, 1∶1000; Sigma-Aldrich), anti-CYB5D2 (1∶200; in-house), and anti-HA (12CA5, 1∶1000), anti-AKT1 (C-20, 1∶1000), anti-β-actin (C-11, 1∶1000), anti-cytochrome P450 reductase (CYPOR) (F-2, 1∶1000), anti-cyclin D1 (H-295, 1∶200), anti-GRP78 (N-20, 1∶200), anti-p21 (C-19, 1∶200), anti-p27 (F-8, 1∶200) from Santa Cruz Biotechnology, anti-IκB-α (#9242, 1∶1000), anti-phospho-AKT (Ser473; #4058, 1∶1000), anti-phospho-ERK1/2 (Thr202/Tyr204, #9101, 1∶1000), anti-ERK1/2 (#9102, 1∶1000) from Cell Signaling Technology. Immunoblot signals were detected using anti-mouse-horse radish peroxidase (HRP) (1∶3000, GE Healthcare), anti-rabbit-HRP (1∶3000, GE Healthcare) or anti-goat-HRP (1∶3000, Santa Cruz Biotechnology) secondary antibodies and ECL reagent (GE Healthcare).

### Statistical Analysis

Statistical analysis was performed using Student’s *t*-test (two-tailed independent), and *p*<0.05 was considered statistically significant.

## Results

### Examination of the cyt-b5 Domain of CYB5D2

**Figure 1:**
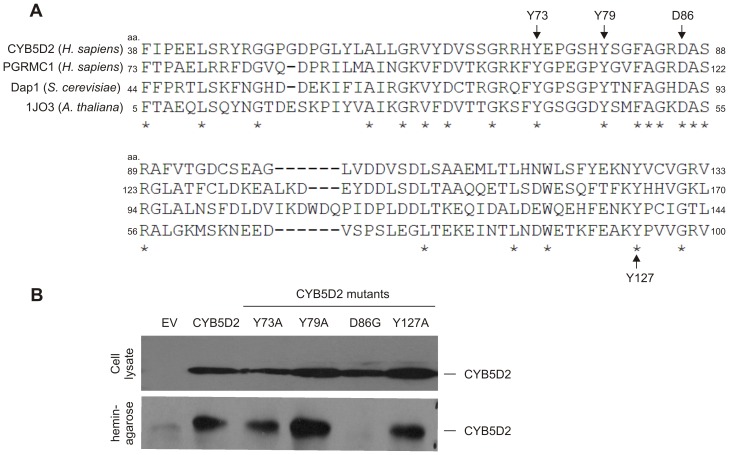
The D86 residue of CYB5D2 contributes to CYB5D2-mediated heme-binding. A) Alignment of the cyt-b5 domain of the human cytochrome b5 domain containing 2 (CYB5D2) protein (amino acid residues (aa.) 38 to 133), human progesterone receptor membrane component 1 (PGRMC1; aa. 73 to 170), damage-associated protein 1 (Dap1) in *Saccharomyces cerevisiae* (aa. 44 to 144), and the 1J03 protein (*Arabidopsis thaliana*; aa. 5 to 100) was performed using Clustal Omega (1.2.0). Identical residues are indicated (*). The D86 residue of CYB5D2 and the conserved D120 residue in PGRMC1 are indicated (arrow), along with conserved tyrosine residues (Y73, Y79, Y127 in CYB5D2) previously implicated in PGRMC1 and Dap1 mediated heme-binding [6]–[8], [12], [25]. B) 293T cells were transiently transfected with empty vector (EV), CYB5D2, and the indicated CYB5D2 mutants. The expression of the ectopic proteins in whole cell lysates was examined by Western blot (top panel), and the ectopic proteins were precipitated by hemin-agarose (bottom panel). Blots were probed with anti-FLAG (M2) antibody.

While CYB5D2 shares 19.8% identity and 26.2% similarity with PGRMC1, the homologous region is largely centered in their cyt-b5 domains which are 40.4% identical and 55.8% similar [10]. Consistent with the well-documented property of the cyt-b5 motif in heme association [1], CYB5D2 (Neuferricin) was reported to bind heme [9]. Whether this association is mediated through specific residues in the cyt-b5 domain of CYB5D2 remains unclear. Upon structural analysis of the 1J03 protein (homolog of the mammalian membrane-associated progesterone receptor) in *Arabidopsis thaliana* [23], [24], it was revealed that key residues and structural elements within its cyt-b5 domain are conserved with PGRMC1 [2]. In researching residues responsible for heme-binding in this protein family, Y107 and Y113 residues in human PGRMC1, as well as the Y138 residue in Dap1 (the yeast homolog of PGRMC1), contribute to heme-binding [8], [12], [25]. These three tyrosine residues are conserved in CYB5D2 as Y73, Y79, and Y127, respectively, as determined by Clustal Omega (1.2.0) alignment (Figure 1A). Additionally, the D120 residue of PGRMC1 was recently reported to play a major role in heme-binding [7]. This critical heme-binding D120 residue of PGRMC1 is conserved at D86 in CYB5D2 (Figure 1A). Furthermore, these four residues are all conserved in 1J03 protein. Collectively, our analysis reveals that amino acid residues Y73, Y79, D86, and Y127 in human CYB5D2 are conserved in human PGRMC1, yeast Dap1, and the 1J03 protein of *Arabidopsis thaliana* (Figure 1A).

### Characterization of CYB5D2-mediated Heme Association

As these conserved residues (Y73, Y79, D86, and Y127) have been reported to contribute to heme association in PGRMC1 and Dap1, we hypothesized that they may also play a role in CYB5D2-mediated heme-binding. To examine this possibility, we individually replaced these three tyrosine residues with alanine (Y73A, Y79A, and Y127A), and the conserved D86 with glycine (D86G). Substitution of glycine (G) at D86 in CYB5D2 was carried out in order to generate a CYB5D2(D86G) mutant since D120G substitution in PGRMC1 [7] and D91G in Dap1 [6] ablated heme-binding in these respective proteins. When transiently expressed in 293T cells, these CYB5D2 mutants were expressed at comparable levels to wild-type CYB5D2 in whole cell lysates (Figure 1B), suggesting that the overall configuration and folding was not significantly affected by the aforementioned substitutions. While CYB5D2, as well as Y73A, Y79A, and Y127A mutants were effectively precipitated by hemin-agarose, the CYB5D2(D86G) mutant was not precipitated (Figure 1B, bottom panel). This result demonstrates that D86 is a critical residue responsible for CYB5D2’s ability to bind to heme.

CYB5D2 contains two conserved motifs: a single transmembrane motif and a cyt-b5 domain [9]. CYB5D2 mutants with deletion of either the transmembrane domain (TM) or cyt-b5 domain were previously generated [10]. To examine the contribution of the TM and cyt-b5 domains towards the heme-binding ability of CYB5D2, these mutants were first successfully expressed in 293T cells (Figure S1, top panel). These mutants were subsequently precipitated with hemin-agarose (Figure S1, bottom panel). The CYB5D2(ΔTM) mutant was able to precipitate with hemin-agarose, demonstrating its competency in binding heme. However, the CYB5D2(Δcyt-b5) mutant could not precipitate with hemin-agarose (Figure S1, bottom panel). Taken together, these observations demonstrate that the cyt-b5 domain, and more importantly the D86 residue within this domain, is critical for heme-binding.

### D86 Residue of CYB5D2 is Required for Heme-binding

**Figure 2:**
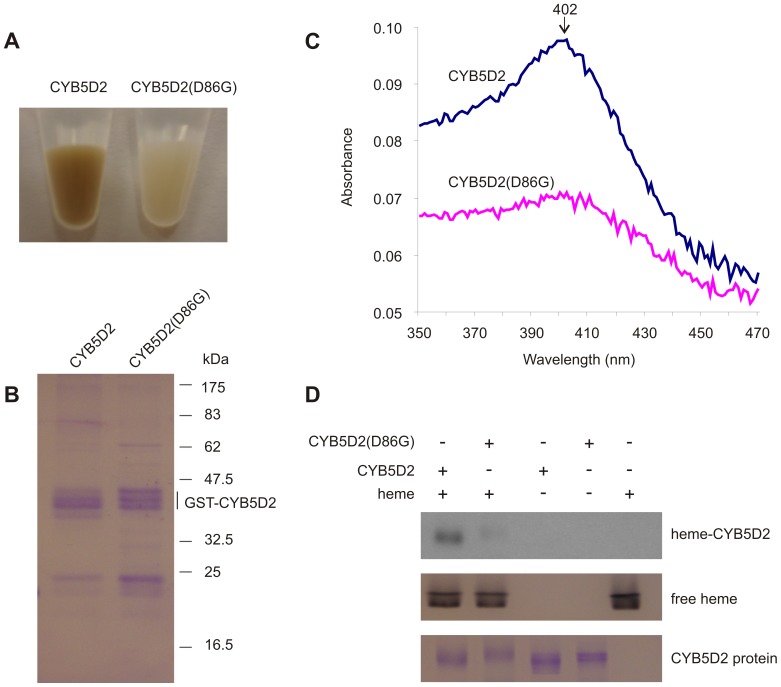
D86G substitution renders recombinant CYB5D2 incapable of binding heme. A) Images of GST-CYB5D2 (CYB5D2)-bound and GST-CYB5D2(D86G)-bound glutathione-agarose. B) 10 µg of purified recombinant GST-CYB5D2 (CYB5D2) and GST-CYB5D2(D86G) (CYB5D2(D86G)) was separated by SDS-PAGE, followed by Coomassie blue staining. C) Recombinant GST-CYB5D2 and GST-CYB5D2(D86G) protein, referred to as CYB5D2 and CYB5D2(D86G) respectively, were scanned for their absorbance peaks within the indicated wavelength range. D) Recombinant GST-CYB5D2 (CYB5D2) and GST-CYB5D2(D86G) (10 μg) were incubated with hemin and analyzed by in-gel peroxidase reaction staining to detect heme binding (see Materials and Methods for technical details). Presence of free (unbound) heme was also detected (middle panel). Equal loading of recombinant proteins was verified by Coomassie blue staining (bottom panel).

The heme-binding analysis carried out following transient expression of CYB5D2 and its mutants in 293T cells, coupled with hemin-agarose pull-down analysis, does not exclude the possibility that the D86 residue may mediate CYB5D2 to associate with a potential heme-binding protein. To eliminate this possibility, we purified GST-CYB5D2 and GST-CYB5D2 (D86G) recombinant fusion proteins using the BL-21 *E. coli* host. To enhance the solubility of these fusion proteins, the transmembrane domain was deleted (ΔTM). During the purification process, we observed that GST-CYB5D2-bound glutathione-agarose displayed a darker brown color compared to GST-CYB5D2(D86G)-bound glutathione-agarose (Figure 2A), an observation consistent with those previously reported for recombinant murine Neuferricin (CYB5D2) and human PGRMC1 proteins [7], [9]. From the respective glutathione-agarose pull-down, GST-CYB5D2 and GST-CYB5D2(D86G) recombinant proteins were subsequently eluted with comparable purity (Figure 2B). Heme-associated proteins possess a maximal absorbance at approximate 402 nm wavelength [7], [9]. In accordance with this property, GST-CYB5D2 protein, but not GST-CYB5D2(D86G), displayed a peak absorbance at approximately 402 nm (Figure 2C). This observation further confirmed that GST-CYB5D2 protein associates with heme and that substitution of D86 with glycine substantially reduced the heme association.

To further consolidate the above observation, we performed in-gel peroxidase reaction staining. When loaded with heme, heme-binding proteins can be detected by peroxidase reaction staining [9], [16], [17]. While recombinant GST-CYB5D2(D86G) protein was not detected by peroxidase reaction staining, with and without heme loading, GST-CYB5D2 protein was detected only under the heme-loaded condition (Figure 2D). In these reactions, heme was present in excess, as comparable amounts of free heme was observed among the reactions of GST-CYB5D2+ heme, GST-CYB5D2(D86G)+heme, and heme alone (Figure 2D, middle panel). Therefore, the inability to detect heme-associated GST-CYB5D2(D86G) protein was not caused by an insufficient amount of heme being present.

### CYB5D2 Binds to Type *b* Heme

**Figure 3:**
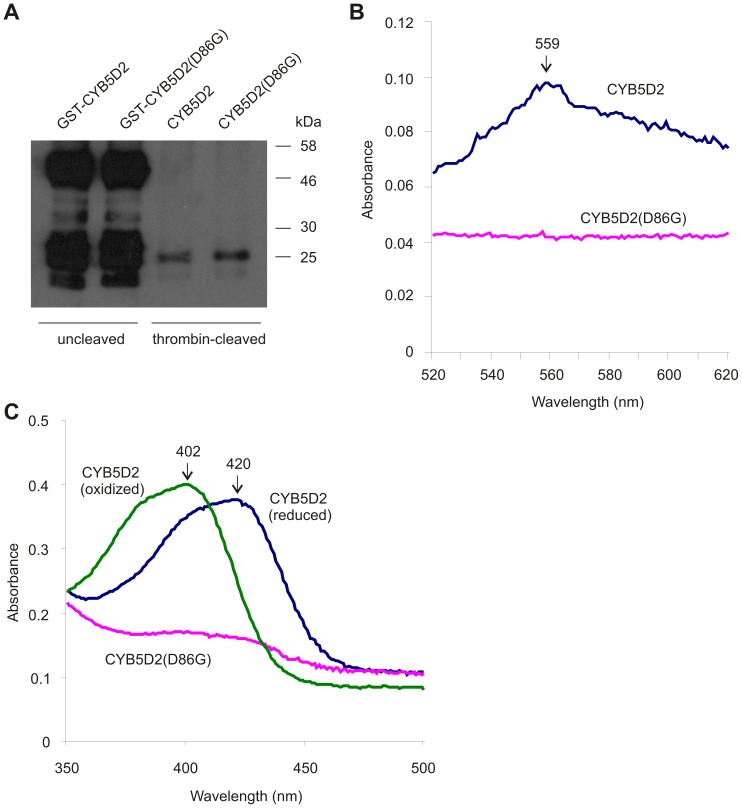
CYB5D2 binds to type *b* heme, and exists in both oxidized and reduced forms. A) Purified recombinant GST-CYB5D2 and GST-CYB5D2(D86G) were thrombin-cleaved, and the uncleaved and cleaved (CYB5D2 and CYB5D2(D86G), respectively) proteins were separated by SDS-PAGE followed by Western blot analysis for CYB5D2 using anti-CYB5D2 antibody. B) Recombinant GST-free CYB5D2 and CYB5D2(D86G) proteins were oxidised by K_3_Fe(CN)_6_, followed by scanning at the indicated wavelength range using a spectrophotometer. The peak absorbance for CYB5D2 was observed at approximately 559 nm. C) Recombinant GST-free CYB5D2 and CYB5D2(D86G) proteins were reduced by sodium dithionite treatment and scanned for their absorbance peaks within the indicated wavelength range. The maximum absorbance peaks for the oxidized and reduced forms of human CYB5D2 were 402 nm and 420 nm, respectively.

The heme population consists of types *a*, *b*, and *c*. While type *c* heme covalently links to proteins (cytochrome *c*), both types *a* and *b* heme attach to protein via non-covalent bonds [19]. In fact, cytochrome *a*, *b*, or *c* were named because of the existence of heme types *a*, *b*, or *c*, respectively. Among the three types of heme, heme *b* is the most common type [19]. Therefore, the existence of a cyt-b5 (heme-binding) motif suggests that CYB5D2 binds type *b* heme. In a reaction containing sodium hydroxide, pyridine and potassium ferricyanide, types *a* and *b* heme associated with heme-binding proteins become oxidized [15]. While type *c* heme does not dissociate from cytochrome *c*, the presence of the protein does not interfere with heme *c* oxidization [15]. Oxidised heme *a*, *b*, and *c* display a specific spectrum of absorbance, with the peak absorbance for types *a*, *b*, and *c* being at 588 nm, 556 nm, and 550 nm, respectively [15]. To exclude the possibility that the presence of GST in the GST-CYB5D2 fusion may affect its association with heme and its subsequent oxidation, the GST portion of the recombinant proteins was cleaved off by treating with thrombin (Figure 3A). When the absorbance of CYB5D2-associated heme was measured, its peak absorbance was observed at approximately 559 nm. Under the same conditions, CYB5D2(D86G) protein did not generate an absorbance peak (Figure 3B). Taken together, these observations suggest that CYB5D2 binds to type *b* heme.

To further characterize the association between CYB5D2 and heme, the absorbance of thrombin-cleaved CYB5D2 and CYB5D2(D86G) recombinant proteins were examined under oxidized and reduced conditions. The GST-free CYB5D2 recombinant protein displayed peak absorbance at 402 nm (oxidized condition) which shifted to 420 nm under reduced conditions (Figure 3C), the typical absorbance pattern for heme-associated proteins including murine CYB5D2 (Neuferricin) [9]. The GST-free CYB5D2(D86G) recombinant protein could not be oxidized or reduced (Figure 3C). Taken together, the above observations reveal that the D86 residue, present within the cyt-b5 domain, is directly responsible for CYB5D2 to bind heme.

### Heme-binding Contributes to CYB5D2-mediated Regulation of HeLa Cell Proliferation and Anchorage-independent Growth

**Figure 4:**
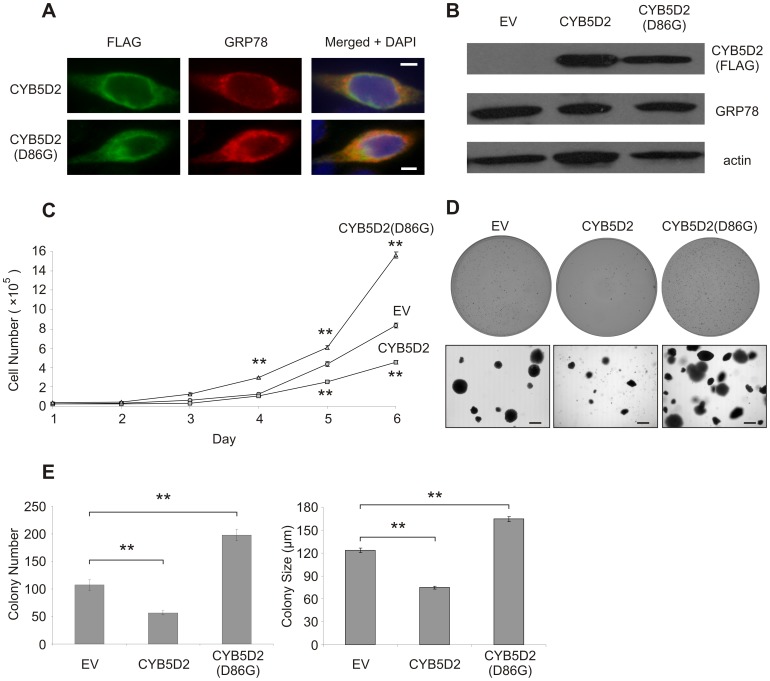
CYB5D2 regulates adherent cell proliferation and colony growth of HeLa cells in a heme-association dependent manner. A) HeLa cells were infected with pLHCX (empty vector; EV), FLAG-CYB5D2/pLHCX, or and FLAG-CYB5D2(D86G)/pLHCX retrovirus as indicated to generate stable cell lines. Dual immunofluorescence of ER-localized GRP78 protein with FLAG-tagged CYB5D2 or CYB5D2(D86G). Images were taken at 1000X magnification. Scale bar is equal to 5 μm. B) The expression of ectopic CYB5D2 and CYB5D2(D86G) proteins in whole cell lysates demonstrated by Western blot analysis. The expression of GRP78 and actin were also determined. C) Cell proliferation of HeLa cells following ectopic expression of CYB5D2 and CYB5D2(D86G). The indicated cell lines were seeded at 10^4^ cell/well in 12-well plates. Cell numbers were determined daily for a period of 6 days. Experiments were conducted in quadruplicate, with triplicate replicates in each experiment. Cell numbers determined at each time point are presented as mean ± SD. **p<0.01 (two-tailed Student’s *t*-test). D) Anchorage-independent growth of CYB5D2 and CYB5D2(D86G)-expressing HeLa cells. Representative images of soft agar plates (top panels) and phase contrast images of colonies at 50X magnification (bottom panels). Scale bar is equal to 200 µm. E) Mean number (left panel) and mean diameter (right panel) of colonies in 10 random fields are presented as mean ± S.E.M. of three independent experiments. **p<0.01 (two-tailed Student’s *t*-test).

In order to examine the function of CYB5D2 and CYB5D2(D86G) in cell culture, stable HeLa cell lines of empty vector (EV) or ectopically-expressing FLAG-tagged CYB5D2 and CYB5D2(D86G) were generated. CYB5D2 has previously been demonstrated to display perinuclear localization in HeLa cells [10]. To reaffirm this observation, we examined the cellular localization of ectopic CYB5D2 and CYB5D2(D86G) proteins. Both CYB5D2 and CYB5D2(D86G) co-localize with the ER protein, GRP78 (Figure 4A), suggesting that modifying CYB5D2’s heme-binding ability does not affect its ER localization. To confirm stable expression of CYB5D2 and CYB5D2(D86G) in HeLa cells, whole cell lysates were prepared and Western blot analysis was performed (Figure 4B). Ectopic CYB5D2 or CYB5D2(D86G) expression in HeLa cells did not induce the unfolded protein response (UPR) as neither CYB5D2 or CYB5D2(D86G) overexpression affected GRP78 protein levels (Figure 4B), an ER chaperone protein upregulated during ER stress [26]–[28]. This suggests that the substitution of D86 with glycine within CYB5D2 does not result in protein misfolding.

CYB5D2 (Neuferricin) has been shown to promote neuron differentiation via inhibiting cell proliferation [9]. To examine the impact of heme-binding of CYB5D2 on cell proliferation in HeLa cells, cell growth curves were conducted. In comparison to EV (control) cells, ectopic CYB5D2 expression reduced HeLa cell proliferation (Figure 4C). However, this inhibition of HeLa cell proliferation is cell density dependent, as seeding at densities <10^4^ cells/well does not result in a difference in cell growth between CYB5D2-expressing cells and EV cells (data not shown). Moreover, the concept that ectopic CYB5D2 reduces HeLa cell proliferation was strengthened by the observation that ectopic expression of the heme-binding defective CYB5D2(D86G) mutant increased cell proliferation (Figure 4C). Therefore, heme-binding by CYB5D2 is required for its ability to regulate HeLa cell proliferation.

The propagation of tumorigenic cells in soft agar is an *in vitro* substitute test of tumorigenicity, as cell growth under anchorage-independent conditions closely associates with *in vivo* tumorigenicity in immunocompromised mice [29]–[31]. To address whether heme-binding by CYB5D2 contributes to HeLa cell anchorage-independent growth, CYB5D2 and CYB5D2(D86G)-expressing HeLa cells were seeded in soft agar-containing, serum-supplemented media. CYB5D2 expression reduced HeLa cell anchorage-independent growth in soft agar, while CYB5D2(D86G) increased colony growth (colony number and size) compared to EV cells (Figure 4D,E). Taken together, heme-binding by CYB5D2 is necessary for inhibiting HeLa cell proliferation, as well as growth under anchorage-independent conditions.

**Figure 5:**
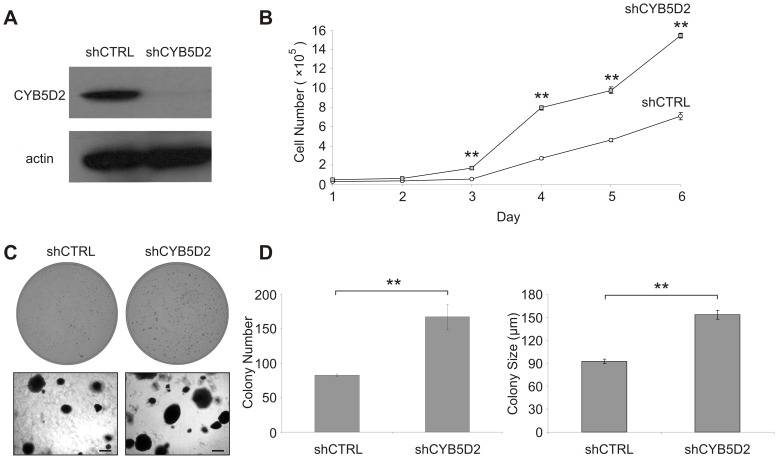
CYB5D2 knockdown promotes adherent cell proliferation and anchorage-independent growth of HeLa cells. A) Western blot analysis of HeLa cells following CYB5D2 shRNA-mediated knockdown (shCYB5D2) compared to shRNA control (shCTRL) HeLa cells. B) Cell proliferation of HeLa cells following CYB5D2 knockdown. Experiments were conducted in quadruplicate, with triplicate replicates in each experiment. Cell numbers determined at each time point are presented as mean ± SD. **p<0.01 (two-tailed Student’s *t*-test). C) Anchorage-independent growth of shCYB5D2 and shCTRL HeLa cells. Representative images of soft agar plates (top panels) and phase contrast images of colonies at 50X magnification (bottom panels). Scale bar is equal to 200 µm. D) Mean number (left panel) and mean diameter (right panel) of colonies in 10 random fields were evaluated and presented as mean ± S.E.M. of three independent experiments. **p<0.01 (two-tailed Student’s *t*-test).

The observation that ectopic CYB5D2(D86G) significantly enhanced HeLa cell proliferation (Figure 4C) and colony growth (Figure 4D,E) suggests that CYB5D2(D86G) may function to inhibit endogenous CYB5D2 function in a dominant-negative fashion. This is supported by the observation that stable shRNA-mediated knockdown of endogenous CYB5D2 enhanced HeLa cell proliferation in comparison to control shRNA (shCTRL) cells (Figure 5A,B). In addition, knockdown of endogenous CYB5D2 in HeLa cells increased their anchorage-independent growth, in terms of colony number and size in soft agar, compared to shCTRL cells (Figure 5C,D). Collectively, these results reveal that the heme-binding activity of CYB5D2 is required for CYB5D2 to inhibit HeLa cell growth under anchorage-dependent and independent conditions.

### CYB5D2 Regulates CYP51A1 Protein Levels, CYP3A4 Activity and Confers Survival from Chemotherapeutic Agents

**Figure 6:**
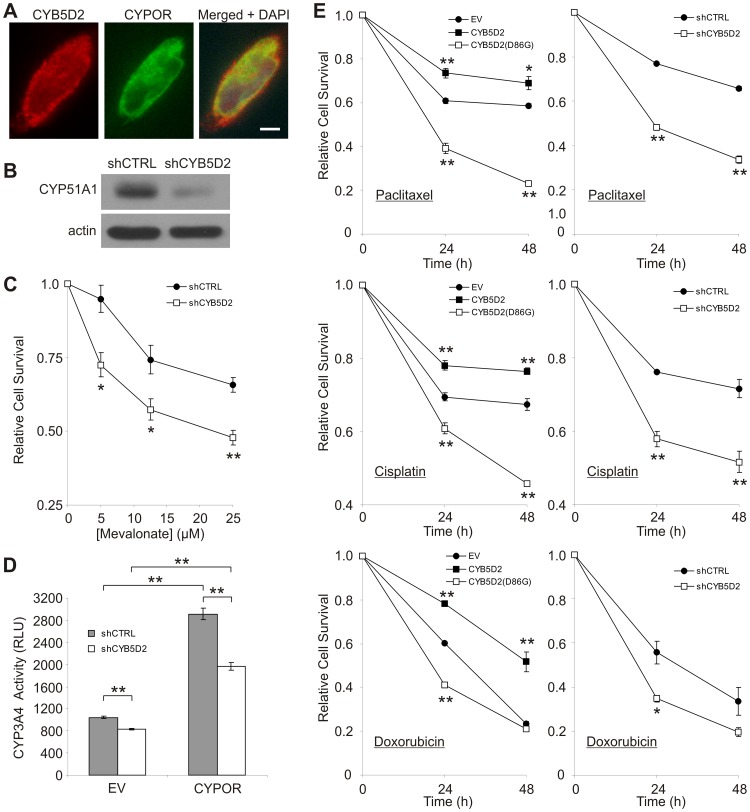
CYB5D2 modulates CYP51A1 protein levels and CYP3A4 activity, with loss of CYB5D2 function rendering HeLa cells sensitive towards chemotherapeutic agents. A) Dual immunofluorescence of FLAG-tagged CYB5D2 and CYPOR-YFP. Stable CYB5D2-expressing HeLa cells were transiently transfected with CYPOR-YFP plasmid. Images were taken at 1000X magnification. Scale bar is equal to 5 μm. B) Western blot analysis of CYP51A1 protein levels following CYB5D2 shRNA-mediated knockdown (shCYB5D2) in HeLa cells compared to shRNA control (shCTRL) cells. C) Mevalonate treatment results in reduced survival of shCYB5D2 cells compared to shCTRL cells. D) CYP3A4 activity is reduced in shCYB5D2 cells compared to shCTRL cells, which is further evident following transiently transfecting CYPOR-YFP plasmid (+CYPOR) for 24 hours (h). E) Relative survival of HeLa cells expressing ectopic CYB5D2 or CYB5D2(D86G) compared to empty vector (EV) control cells (left panels), or shRNA control (shCTRL) and CYB5D2 shRNA-mediated knockdown (shCYB5D2) HeLa cells (right panels), following treatment with either paclitaxel (400 ng/ml), cisplatin (400 ng/ml) or doxorubicin (10 µM) for 24 and 48 h. At each time point, cell survival values were normalized to a dimethylsufloxide (DMSO)-treated control for each stable cell line. Relative cell survival values are presented as mean ± S.E.M. of three independent experiments (three replicates in each experiment). *p<0.05; **p<0.01 (two-tailed Student’s *t*-test).

PGRMC1 and its *S. cerevisiae* homolog, Dap1, interact with and regulate cytochrome P450 (CYP) proteins [6], [12], enzymes which are involved in xenobiotic detoxification, pharmaceutical drug metabolism, as well as sterol and steroid biosynthesis [11]. Furthermore, PGRMC1 has been shown to co-localize and bind with cytochrome P450 reductase (CYPOR) [13], a redox partner required by all microsomal cytochrome P450 monooxygenases for their catalytic activities [32]. Similarly, CYB5D2 co-localizes with ectopic CYPOR protein in HeLa cells (Figure 6A), suggesting that CYB5D2 may associate with CYPOR and modulate CYP activities.

Dap1 has been shown to regulate and stabilize yeast *Cyp51A1* [4], [6], while human PGRMC1 has been shown to bind to CYP51A1 (lanosterol demethylase) and modulate its activity [12], [13]. Therefore, we examined whether the endogenous levels of CYP51A1 protein were affected following CYB5D2 knockdown in HeLa cells. As shown in Figure 6B, CYB5D2 knockdown resulted in decreased CYP51A1 protein levels compared to shCTRL cells. Consistent with Dap1 regulating *Cyp51A1* stability and activity, yeast lacking Dap1 had higher levels of lanosterol and squalene [6]. Similarly, PRGMC1 knockdown resulted in elevated lanosterol levels in HEK293 cells [12], [13]. This defect was evident following treatment of PGRMC1 knockdown cells with mevalonate, which exposed their susceptibility to an increase in the sterol synthesis pathway [12], [13]. Mevalonate is the first intermediate produced in a series of enzymatic reactions leading to cholesterol synthesis [33]. Therefore, the survival capacity of CYB5D2 knockdown in HeLa cells was examined following treatment with increasing mevalonate concentrations under lipoprotein-free conditions. As shown in Figure 6C, CYB5D2 knockdown increased the susceptibility of HeLa cells towards increasing mevalonate concentrations, supporting a defect in the lanosterol demethylase step within the mevalonate pathway.

CYP3A4, one of the most abundantly expressed cytochromes in human liver with a broad substrate selectivity [34], plays a major role in the metabolism of greater than 30% of clinical drugs [35], [36] including paclitaxel [37], cisplatin [38] and doxorubicin [39]. In PGRMC1-deficient cells, the enzymatic activity of CYP3A4 has previously been shown to be slightly higher compared to control cells [13]. Upon examining the role of CYP5D2 in regulating CYP3A4 enzymatic activity, a decrease in CYP3A4 activity was observed following CYB5D2 knockdown in HeLa cells. This reduction is further evident following transient transfection of CYPOR (Figure 6D). While ectopically-transfected CYPOR increased CYP3A4 activity in shCTRL cells, this activity was significantly higher than the CYP3A4 activity levels observed in CYB5D2 knockdown cells (Figure 6D). Taken together, CYBD2 co-localizes with CYPOR and is required for modulating CYP51A1 protein levels and CYP3A4 activity in HeLa cells.

Since a number of CYP proteins are involved in drug metabolism and xenobiotic detoxification [11], we investigated whether loss of CYB5D2 function sensitizes HeLa cells towards chemotherapeutic drug treatment. CYB5D2 has been shown to enhance HeLa cell survival from etoposide [10]. As shown in Figure S2, HeLa cells expressing ectopic CYB5D2 were more resistant to increasing concentrations of paclitaxel (mitotic inhibitor) and cisplatin (DNA cross-linking inducer) treatment, using a concentration range which has previously been shown to be effective at reducing HeLa cell viability after 48 h treatment [40], [41]. Furthermore, CYB5D2(D86G) expression resulted in increased susceptibility towards paclitaxel and cisplatin treatment in a dose-dependent fashion (Figure S2). These dose response effects were clearly dramatic at a concentration of 400 ng/ml for both paclitaxel and cisplatin (Figure S2). Therefore, the sensitivity of stable HeLa cell lines towards paclitaxel or cisplatin was evaluated at this concentration following 24 and 48 h treatments (Figure 6E). Treatment of CYB5D2-expressing HeLa cells with either paclitaxel or cisplatin resulted in enhanced cell survival relative to EV cells in a time-dependent fashion (Figure 6E). Conversely, the CYB5D2(D86G) HeLa cells demonstrated an increased susceptibility to paclitaxel and cisplatin after 24 and 48 h, relative to EV cells (Figure 6E). Furthermore, CYB5D2 knockdown resulted in reduced HeLa cell viability following paclitaxel and cisplatin treatments relative to shCTRL cells (Figure 6E). Lastly, treatment of these stable HeLa cell lines with doxorubicin (topoisomerase II inhibitor) (10 μM) demonstrated that CYB5D2 expression conferred greater cell survival, while loss of function (CYB5D2(D86G) expression and CYB5D2 knockdown) reduced cell survival relative to control cells (Figure 6E, bottom panels). This doxorubicin concentration was consistent with a ∼75% reduction in HeLa cell viability after 48 h treatment [42]. Taken together, these results demonstrate that CYB5D2 confers HeLa cell survival from chemotherapeutic agents, which is dependent on its ability to bind heme.

### Heme-binding is Required for Ectopic CYB5D2 Secretion in 293T Cells

It has recently been reported that murine CYB5D2 (Neuferricin) can be secreted from Neuro2a neuroblastoma cells, with Neuferricin knockdown promoting cell survival, proliferation and inhibiting neurite outgrowth [9]. In addition, exogenous recombinant Neuferricin, but not Neuferricin(ΔHBD) (deletion of heme-binding domain), suppressed cell survival in a dose-dependent fashion [9]. Therefore, we examined the secretion of ectopic CYB5D2 and CYB5D2(D86G) in HeLa cell conditioned media, however, the ectopic proteins could not be detected as being secreted by Western blot analysis (data not shown). This suggests that secretion of CYB5D2 is cell line specific, and implicates its intracellular role in mediating its proliferative and survival functions in HeLa cells (Figures 4, 5 and 6). However, when transiently expressed in 293T cells, CYB5D2, but not CYB5D2(D86G), was readily detected in conditioned media (Figure S3A, top panel), even though both ectopic CYB5D2 and CYB5D2(D86G) proteins were expressed at comparable levels in whole cell lysates (Figure S3A, middle and bottom panels). This suggests that binding to heme is required for the secretion of ectopic CYB5D2 protein from 293T cells.

We subsequently investigated whether the structural elements present in CYB5D2 (TM, cyt-b5), affect its secretion. Upon transient transfection into 293T cells, the CYB5D2(D86G) and CYB5D2(Δcyt-b5) mutants were expressed at comparable levels to wild-type CYB5D2. However, the CYB5D2(ΔTM) mutant was expressed at an increased level relative to the other mutants (Figure S3B, middle panel). In comparison to positive (CYB5D2) and negative [CYB5D2(D86G)] controls, CYB5D2(Δcyt-b5) protein was not detected in the conditioned medium, while the CYB5D2(ΔTM) mutant was detected in its respective conditioned medium (Figure S3A, top panel). Therefore, the CYB5D2(D86G) and CYB5D2(Δcyt-b5) mutants, which are unable to bind heme (Figures 1 and S1), are incapable of being secreted from 293T cells (Figure S3). These observations reveal that CYB5D2 ectopically expressed in 293T cells can be secreted, as long as it is capable of binding heme.

## Discussion

CYB5D2 (Neuferricin) is a heme-binding protein [9], which binds to type *b* heme. Furthermore, CYB5D2 contains a cyt-b5 domain [9], which shares homology with other cyt-b5 domain containing proteins (Figure 1) and is required for CYB5D2 to associate with heme [9]. In the current study, we demonstrate that the D86 residue, a highly conserved amino acid within the cyt-b5 region, is required for CYB5D2 to bind heme. This is based on our results showing that CYB5D2, but not CYB5D2(D86G), binds heme when expressed in mammalian cells and when purified as a recombinant protein from *E. coli* (Figures 1, 2 and 3).

CYB5D2 belongs to the family of membrane-associated progesterone receptors (MAPRs) which possess a cyt-b5 domain [43]. MAPRs affect multiple cellular processes, including proliferation, differentiation, and survival from genotoxic agents [1], [2], [43]. Furthermore, CYB5D2 regulates neural cell growth and survival [9]. Our research establishes a functional connection between heme-binding of CYB5D2 and HeLa cell growth under anchorage-dependent and independent conditions. Ectopic CYB5D2 expression reduced HeLa cell proliferation and colony growth, while expression of the CYB5D2(D86G) mutant or CYB5D2 knockdown increased their proliferative capacity and growth in soft agar (Figures 4 and 5). As a result, these effects suggest that CYB5D2 functions as an inhibitor of cell proliferation, and further implicates that heme-binding is required for its putative tumor suppressor activity. Interestingly, these results are opposite to those reported for PGRMC1 which has been shown to promote MDA-MB-468 breast cancer cell growth in the presence and absence of serum, while the heme-binding defective PGRMC1(D120G) mutant inhibited cell proliferation [44]. Activation of ERK signaling, a prominent signaling pathway in cancer cells [45], remained unaffected following ectopic expression of CYBD2 or CYB5D2(D86G), and following CYB5D2 knockdown (data not shown). This is consistent with results reporting no discernable differences in ERK signaling following CYB5D2 overexpression [9]. In addition, no differences in AKT signal activation were observed, or differences in expression of various cell cycle regulators (cyclin D1, p27 or p21) or total IκB-α protein levels following CYB5D2 knockdown or expression of ectopic CYB5D2 and CYB5D2(D86G) (Figure S4). As similar trends in anchorage-dependent and independent growth were observed following either CYB5D2 knockdown or ectopic CYB5D2(D86G) expression in HeLa cells, loss of CYB5D2 function contributes towards an increase in HeLa cell growth through some undefined molecular mechanism.

The role of cytochrome b5 heme-binding (cyt-b5) domain containing proteins (yeast Dap1 and human PGRMC1) in binding and regulating CYP proteins has been recently investigated [12], [13]. Dap1 was reported to interact with two yeast P450 cytochromes, Cyp51A1 and Cyp61A1 [12]. Additionally, PGRMC1 has been shown to associate with four human microsomal CYP enzymes: CYP51A1, CYP3A4, CYP21A2, and CYP7A1 [12]. PGRMC1 regulates the activity of CYP51A1 (lanosterol demethylase) [12], [13], which is required for sterol production [46], [47]. Furthermore, PGRMC1-deficient cells display higher levels of lanosterol [12], [13], an intermediate of the sterol synthesis pathway [47], and demonstrate increased sensitivity to mevalonate [12], [13]. Similar to Dap1, CYB5D2 knockdown significantly reduces CYP51A1 protein levels, affecting their sensitivity towards mevalonate treatment and implicating CYB5D2 protein in stabilizing CYP51A1 protein levels. Affymetrix DNA microarrays failed to detect any differences in CYP51A1 mRNA levels in CYB5D2-expressing HeLa cells compared to EV HeLa cells [10], supporting that this difference in CYP51A1 protein levels is likely not regulated at the level of transcription. More recently, PGRMC1 was shown to interact with three drug metabolizing P450 cytochromes (CYP2C2, CYP2C8 and CYP3A4) and inhibit their activities [13]. Unlike PGRMC1-deficient cells which display slightly higher CYP3A4 activity levels [13], CYB5D2 knockdown reduced CYP3A4 activity, which was more evident following CYPOR transfection (Figure 6C). Therefore, PGRMC1 and CYB5D2 may specifically modulate the activities of CYP proteins differently.

In conclusion, we provide evidence that binding of heme is required for CYB5D2 to regulate HeLa cell growth and confer its survival from treatment of chemotherapeutic agents, paclitaxel, cisplatin and doxorubicin. Furthermore, this is the first study to report that CYB5D2 regulates the activities of two cytochrome P450 proteins, CYP51A1 and CYP3A4, which are involved in sterol synthesis and drug metabolism, respectively. As it has been previously reported that CYB5D2 does not impact the core processes of apoptosis in HeLa cells [10], it will be intriguing to further decipher the survival mechanisms regulated by CYB5D2. Interestingly, CYB5D2 and PGRMC1 mediate opposing effects on cell proliferation, and possibly other intracellular (ER-localized) functions associated with heme-binding including CYP activities in a CYPOR-mediated fashion. We are currently investigating additional CYP proteins whose activity may be regulated by CYB5D2. Overall, the emerging role of CYB5D2 as a protein that confers survival from chemotherapeutic agents makes it an attractive target for future therapies.

## Supporting Information

Figure S1 **Characterization of CYB5D2-mediated heme-binding.** A) Schematic representation of CYB5D2, CYB5D2(D86G) and mutants with deletions (Δ) of either transmembrane (TM) or cyt-b5 domains, as well as their ability to bind heme. B) Transient expression of the indicated complementary DNA (cDNA) domain deletion mutants in 293T cells (top panel). The heme-binding capacity was analyzed by hemin-agarose precipitation (bottom panel).
(TIF)

Figure S2 **Dose-dependent response curves following treatment of HeLa cells with paclitaxel and cisplatin.** Relative survival of paclitaxel and cisplatin-treated HeLa cells expressing ectopic CYB5D2 or CYB5D2(D86G) compared to empty vector (EV) control cells. Cells were treated with increasing concentrations of paclitaxel or cisplatin for 24 hours (h) (left panels) or 48 h (right panels). Cell survival values were normalized to a dimethylsufloxide (DMSO)-treated control for each stable cell line. Relative cell survival values are presented as *mean ±* S.E.M. of three independent experiments (three replicates in each experiment). *p<0.05; **p<0.01 (two-tailed Student’s *t*-test).
(TIF)

Figure S3 **Heme-binding defective mutants of CYB5D2 cannot undergo cell secretion following ectopic expression in 293T cells.** A) CYB5D2 and CYB5D2(D86G) were transiently expressed for 48 hours in 293T cells. Cell lysates and conditioned medium were analysed by Western blot for CYB5D2 and actin expression. B) The secretion of wild-type CYB5D2 and its domain deletion (ΔTM and Δcyt-b5) mutants were also analyzed by Western blot. Blots were probed with anti-HA antibody. * indicates background bands.
(TIF)

Figure S4 **Examination of cell cycle regulators, IκB-α protein levels, and AKT signal activation in CYB5D2-expressing and CYB5D2 loss-of-function HeLa cells.** Western blot analysis of empty vector (EV), CYB5D2 and CYB5D2(D86G)-expressing HeLa cells (left panels), or shRNA control (shCTRL) and CYB5D2 shRNA-mediated knockdown (shCYB5D2) HeLa cells (right panels). Expression of cyclin D1, p21, p27 and IκB-α proteins were examined. AKT activation was determined by examining the phosphorylation of AKT at the Ser473 residue (AKT-P). Actin was used as the loading control.
(TIF)

Table S1 **Primers and PCR conditions used to construct CYB5D2 mutants.**
(DOC)
