# Supplementary figures and images for: CYB5D2 Requires Heme-Binding to Regulate HeLa Cell Growth and Confer Survival from Chemotherapeutic Agents
Source: PLoS One. 2014 Jan 22;9(1):e86435. doi: 10.1371/journal.pone.0086435 (PMC3899279; doi:10.1371/journal.pone.0086435)

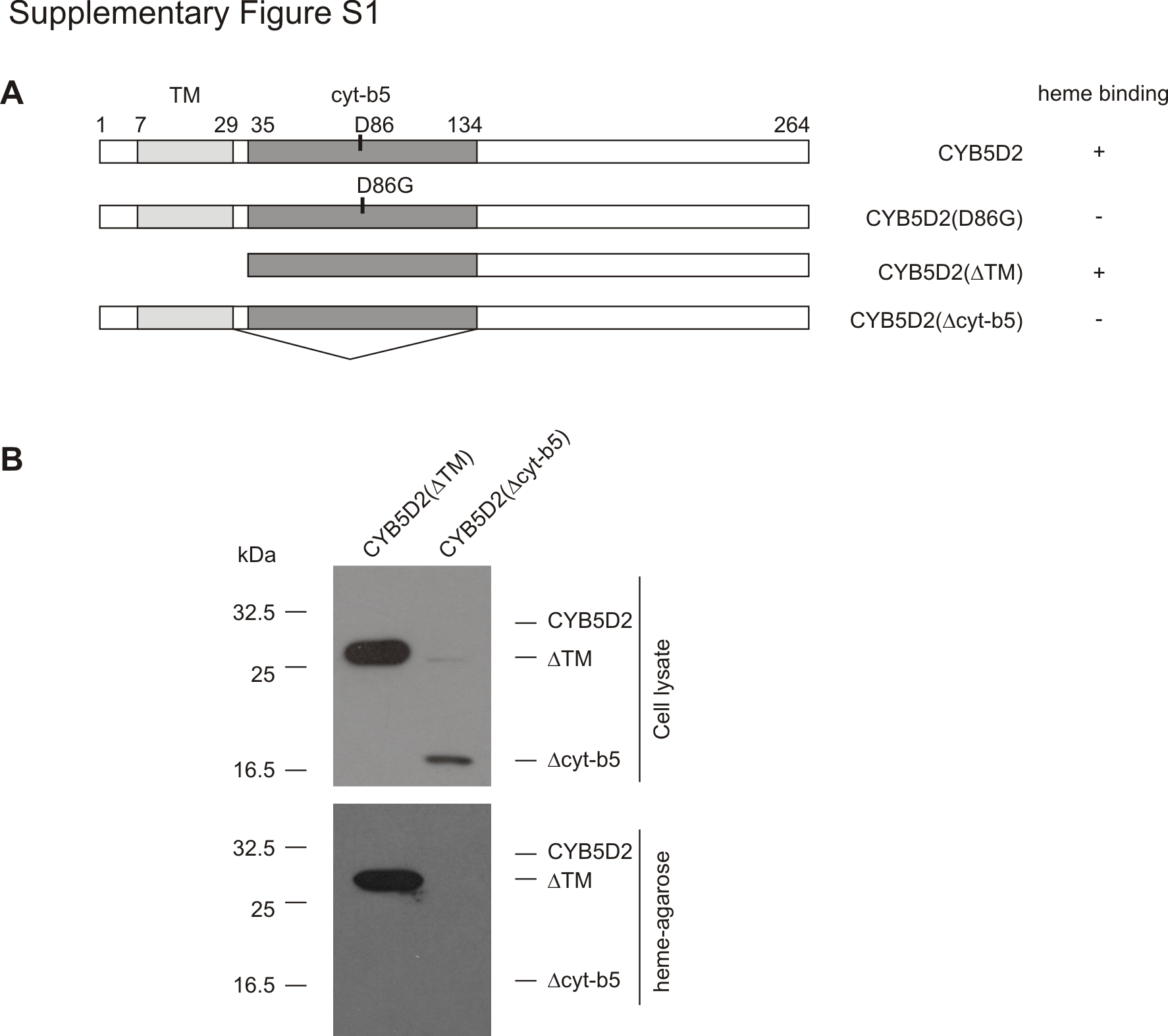

Supplement: Figure S1 — Characterization of CYB5D2-mediated heme-binding. A) Schematic representation of CYB5D2, CYB5D2(D86G) and mutants with deletions (Δ) of either transmembrane (TM) or cyt-b5 domains, as well as their ability to bind heme. B) Transient expression of the indicated complementary DNA (cDNA) domain deletion mutants in 293T cells (top panel). The heme-binding capacity was analyzed by hemin-agarose precipitation (bottom panel). (TIF) [file pone.0086435.s001.tif]

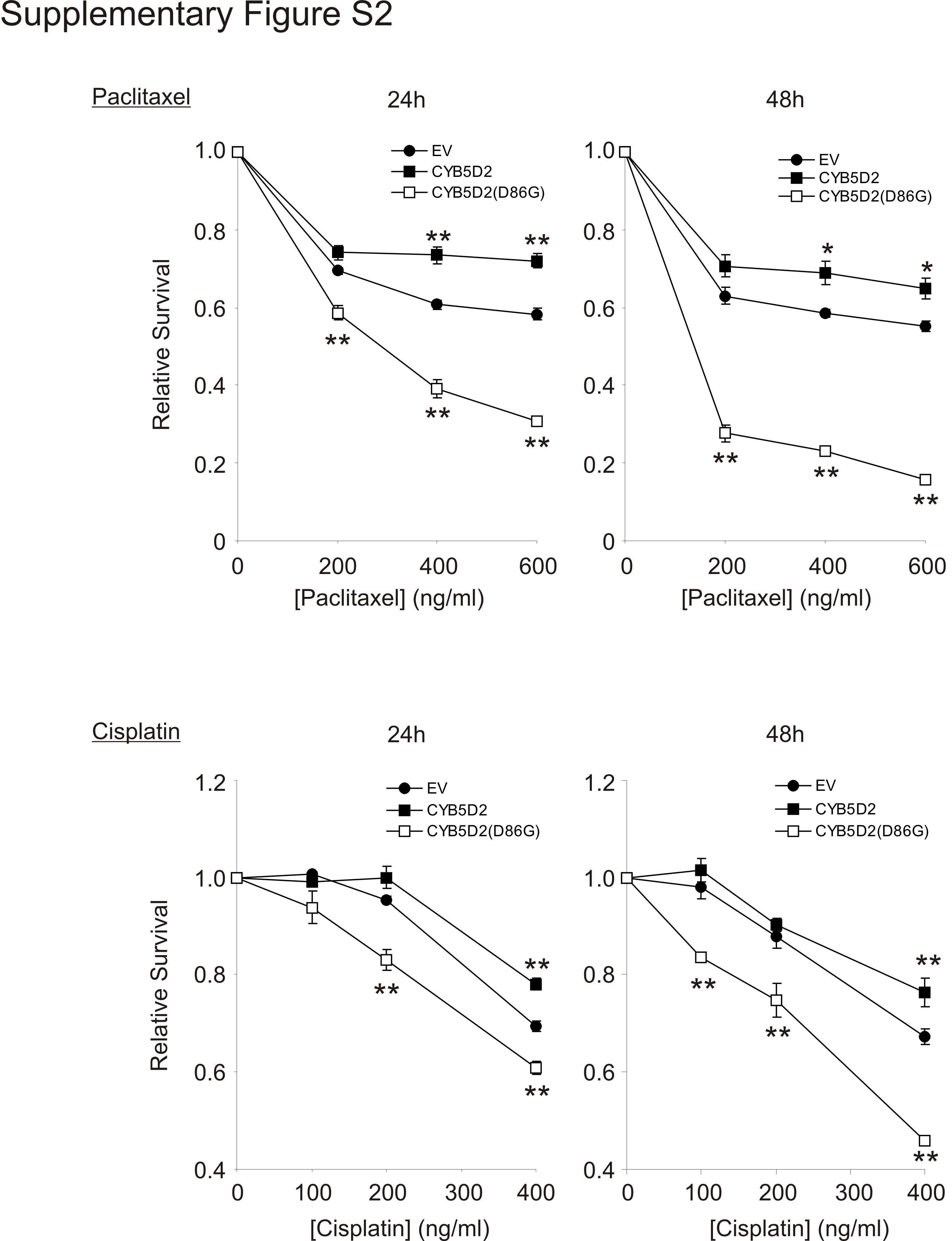

Supplement: Figure S2 — Dose-dependent response curves following treatment of HeLa cells with paclitaxel and cisplatin. Relative survival of paclitaxel and cisplatin-treated HeLa cells expressing ectopic CYB5D2 or CYB5D2(D86G) compared to empty vector (EV) control cells. Cells were treated with increasing concentrations of paclitaxel or cisplatin for 24 hours (h) (left panels) or 48 h (right panels). Cell survival values were normalized to a dimethylsufloxide (DMSO)-treated control for each stable cell line. Relative cell survival values are presented as mean ± S.E.M. of three independent experiments (three replicates in each experiment). *p<0.05; **p<0.01 (two-tailed Student’s t-test). (TIF) [file pone.0086435.s002.tif]

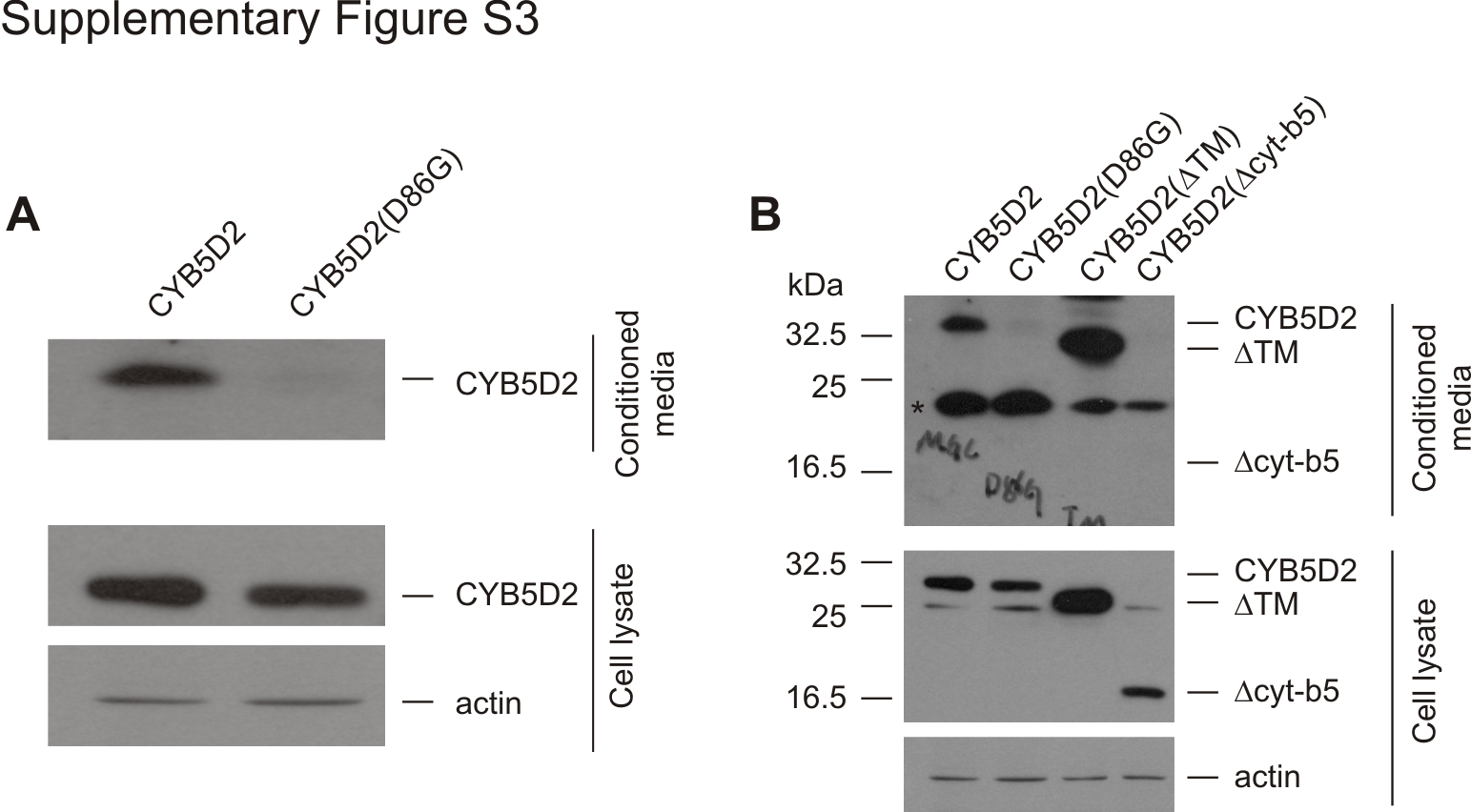

Supplement: Figure S3 — Heme-binding defective mutants of CYB5D2 cannot undergo cell secretion following ectopic expression in 293T cells. A) CYB5D2 and CYB5D2(D86G) were transiently expressed for 48 hours in 293T cells. Cell lysates and conditioned medium were analysed by Western blot for CYB5D2 and actin expression. B) The secretion of wild-type CYB5D2 and its domain deletion (ΔTM and Δcyt-b5) mutants were also analyzed by Western blot. Blots were probed with anti-HA antibody. * indicates background bands. (TIF) [file pone.0086435.s003.tif]

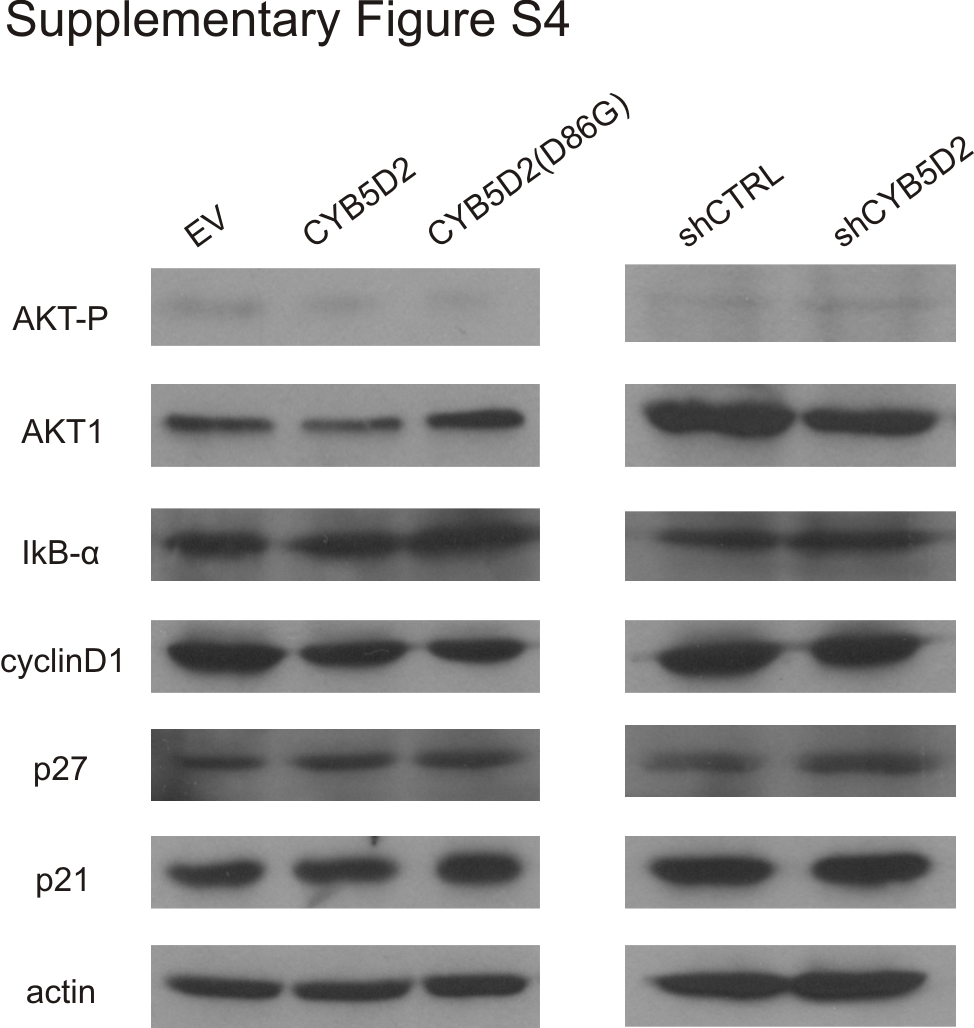

Supplement: Figure S4 — Examination of cell cycle regulators, IκB-α protein levels, and AKT signal activation in CYB5D2-expressing and CYB5D2 loss-of-function HeLa cells. Western blot analysis of empty vector (EV), CYB5D2 and CYB5D2(D86G)-expressing HeLa cells (left panels), or shRNA control (shCTRL) and CYB5D2 shRNA-mediated knockdown (shCYB5D2) HeLa cells (right panels). Expression of cyclin D1, p21, p27 and IκB-α proteins were examined. AKT activation was determined by examining the phosphorylation of AKT at the Ser473 residue (AKT-P). Actin was used as the loading control. (TIF) [file pone.0086435.s004.tif]
