# Supplementary material for: CYB5D2 Requires Heme-Binding to Regulate HeLa Cell Growth and Confer Survival from Chemotherapeutic Agents
Source: PLoS One. 2014 Jan 22;9(1):e86435. doi: 10.1371/journal.pone.0086435 (PMC3899279; doi:10.1371/journal.pone.0086435)
Supplement: Table S1 — Primers and PCR conditions used to construct CYB5D2 mutants. (DOC) [file pone.0086435.s005.doc]

**Table S1.** Primers and PCR conditions used to construct CYB5D2 mutants.

| **Construct** | **Primers (5’ to 3’)** | **PCR Conditions** |
| --- | --- | --- |
| Y73A | Primer 1: GTCCTCCGGCCGGAGGCACTTTGAGCCTGGGTCCCACTATAG  Primer 2: CTATAGTGGGACCCAGGCTCAAAGTGCCTCCGGCCGGAGGAC | 95^o^C 50 seconds, 50^o^C 50 seconds  68^o^C 7 minutes, 18 cycles |
| Y79A | Primer 1: CTGGGTCCCACTTTAGCGGCTTCGC  Primer 2: GCGAAGCCGCTGGCGTGGGACCCAG | 95^o^C 50 seconds, 60^o^C 50 seconds  68^o^C 7 minutes, 18 cycles |
| D86G | Primer 1: CTTCGCAGGCCGAGGAGCATCCAGAGCTTTC  Primer 2: GAAAGCTCTCTGGATGCTCCTCGGCCTGCGAAG | 95^o^C 50 seconds, 60^o^C 50 seconds  68^o^C 7 minutes, 18 cycles |
| Y127A | Primer 1: CATTCTATGAGAAGAATTTTGTGTGTGTTGGGAGGG  Primer 2: CCCTCCCAACACACACAAAATTCTTCTCATAGAATG | 95^o^C 50 seconds, 60^o^C 50 seconds  68^o^C 7 minutes, 18 cycles |
| GST-CYB5D2/  GST-CYB5D2(D86G) | Primer 1: CGGGATCCGGTCCCCGCGCT  Primer 2: GGAATTCTTAGAGTGGAAAGGAGCAT | 94^o^C 45 seconds, 56^o^C 45 seconds  72^o^C 2 minutes, 35 cycles |
